# Correction: Fluorescence activation mechanism and imaging of drug permeation with new sensors for smoking-cessation ligands

**DOI:** 10.7554/eLife.85479

**Published:** 2022-12-15

**Authors:** Aaron L Nichols, Zack Blumenfeld, Chengcheng Fan, Laura Luebbert, Annet EM Blom, Bruce N Cohen, Jonathan S Marvin, Philip M Borden, Charlene H Kim, Anand K Muthusamy, Amol V Shivange, Hailey J Knox, Hugo Rego Campello, Jonathan H Wang, Dennis A Dougherty, Loren L Looger, Timothy Gallagher, Douglas C Rees, Henry A Lester

**Keywords:** Mouse

Nichols AL, Blumenfeld Z, Fan C, Luebbert L, Blom AEM, Cohen BN, Marvin JS, Borden PM, Kim CH, Muthusamy AK, Shivange AV, Knox HJ, Campello HR, Wang JH, Dougherty DA, Looger LL, Gallagher T, Rees DC, Lester HA. 2022. Fluorescence activation mechanism and imaging of drug permeation with new sensors for smoking-cessation ligands. *eLife* **11**:e74648. doi: 10.7554/eLife.74648.

Published 4 January 2022

We now correct an error in Figure 1. “Revision 0” of the paper, submitted on 8 November 2021 and reviewed by the Reviewers 1, 2, and 3, contained the intended, correct version of Figure 1:

**Figure 1:**
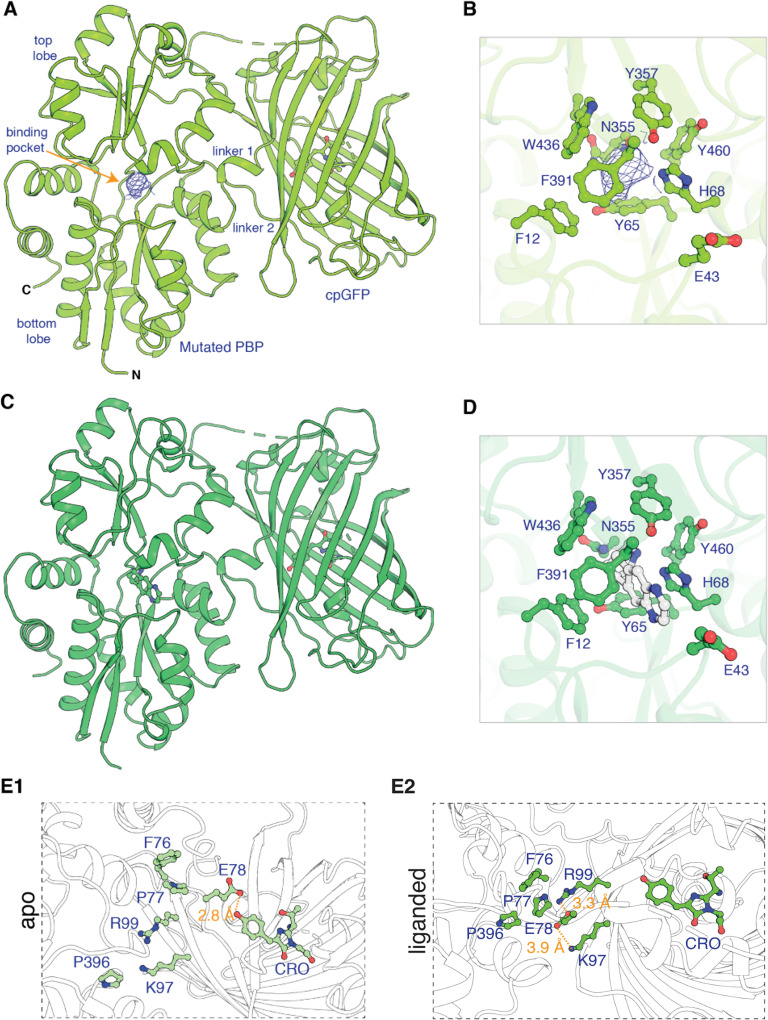


However, the authors made a mistake on “Revision #1”, submitted 30 December 2012 and accepted on 3 January 2022. In that mistake, Figure 1 Panel E2 was duplicated from Figure Panel E1. This mistaken duplication in Panel E2 was part of the originally published version, shown here for reference:

**Figure 2:**
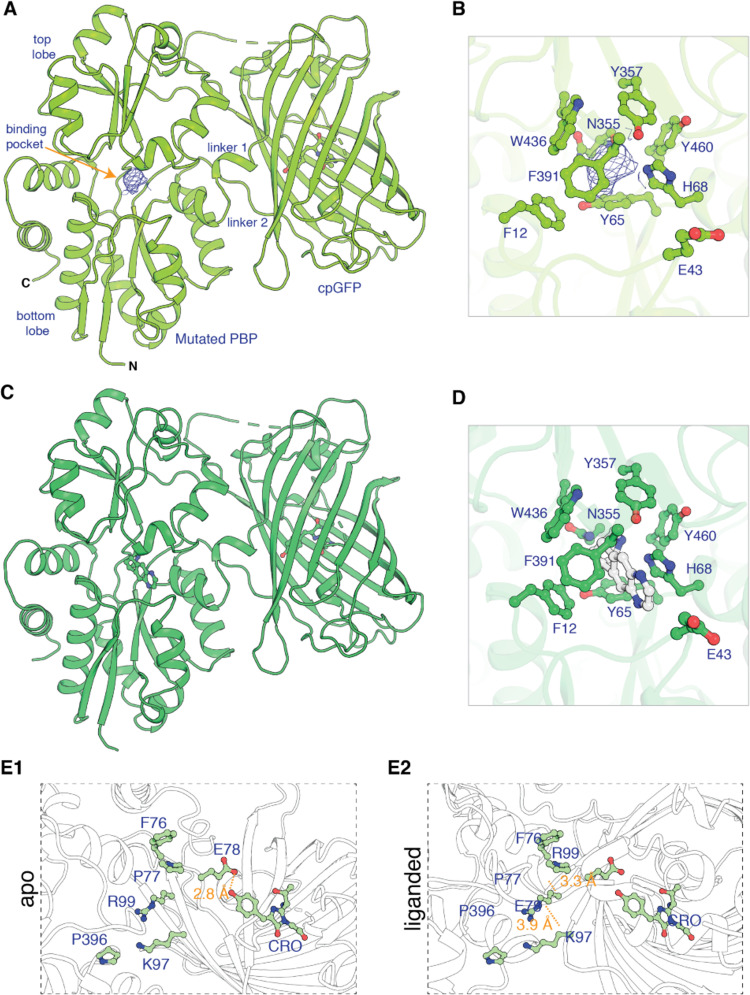


Also, we now correct calculation and presentation errors in the stopped-flow data reported in Figure 5 and in Supplementary File 2.

In Figure 5, the corrected legend for panels (A – D) lists the [drug] values after mixing in the stopped-flow chamber. There was no need to correct the calculations and curve fits (V_max_ and K_d_ values) for panels 5E, F, and G.

In addition, the corrected Figure 5G now includes the previously omitted 10-fluorocytisine point at 500 μM.

The stopped-flow experiments for iCyt_BrEt_SnFR at 31.25 μM and 7.8 μM had different sampling rates than all other measurements. The corrected Figure 5H now presents corrected k_obs_ values for these measurements, leading to corrected, fitted V_max_ and K_d_ for iCyt_BrEt_SnFR.

The corrected Figure 5:

**Figure 3:**
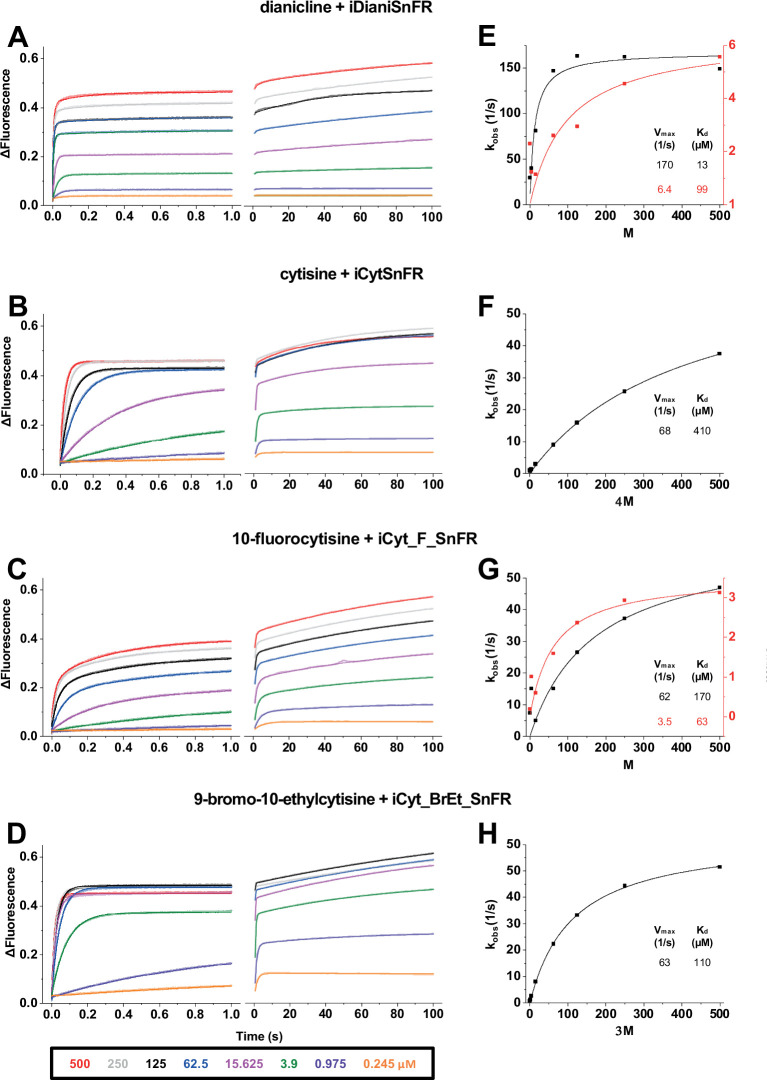


The originally published Figure 5 is shown here for reference:

**Figure 4:**
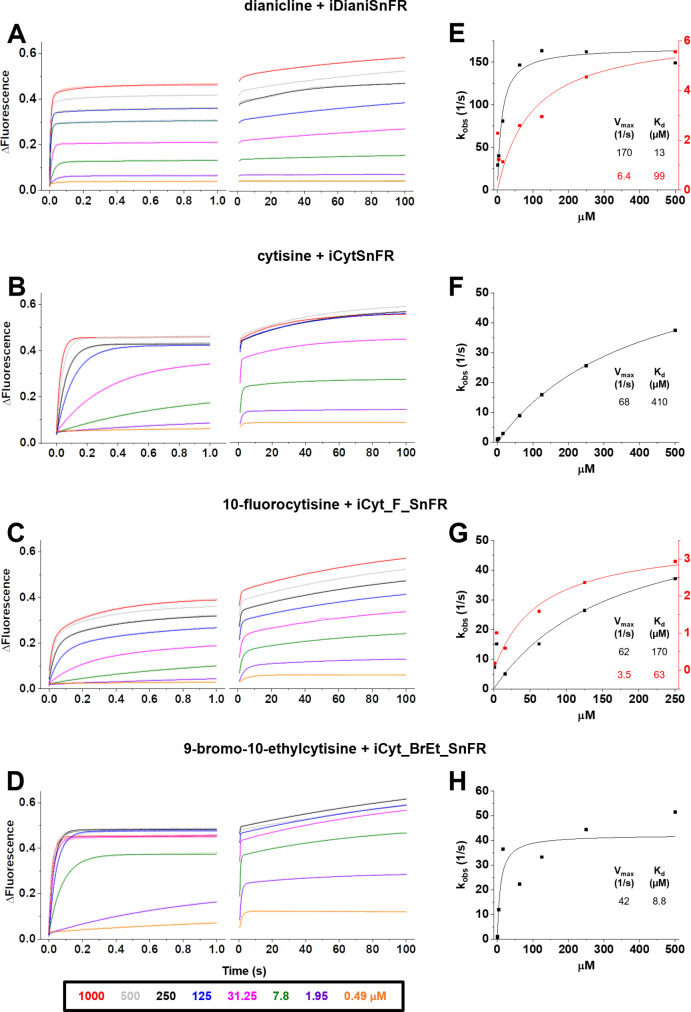


The corrected caption for Figure 5 should now read:

Figure 5. Stopped-flow fluorescence kinetic data for (**A**) iDianiSnFR, (**B**) iCytSnFR, (**C**) iCyt_F_SnFR, and (**D**) iCyt_BrEt_SnFR over 1 s and 100 s.

Fluorescence was activated by mixing with the agonists, producing the indicated final concentrations. Stopped-flow data show a departure from first-order kinetics for this set of iDrugSnFRs. iDianiSnFR and iCyt_F_SnFR are fitted to a double exponential; iCytSnFR and iCyt_BrEt_SnFR are fitted to a single exponential. (**E-H**) Plots of the observed apparent rate constant k_obs_ against [agonist] for the 1 s data obtained in (**A-D**).

The original caption for Figure 5 is shown for reference:

Figure 5. Stopped-flow fluorescence kinetic data for (**A**) iDianiSnFR, (**B**) iCytSnFR, (**C**) iCyt_F_SnFR, and (**D**) iCyt_BrEt_SnFR over 1 s and 100 s.

Fluorescence was activated by mixing with the agonists as noted. Stopped-flow data shows a departure from first-order kinetics for this set of intensity-based drug-sensing fluorescent reporter (iDrugSnFRs). iDianiSnFR and iCyt_F_SnFR are fit to a double exponential; iCytSnFR and iCyt_BrEt_SnFR are fit to a single exponential. (E–H) Plots of the observed apparent rate constant against [agonist] for the 1 s data obtained in (A–D). In (H), we have confidence that the kobs shows a maximal value of 40–50 s–1; the Kd probably lies within twofold of the fitted value.

The corrected caption for Figure 5 removes an ambiguity about the final concentrations. The corrected caption for Figure 5 also removes the final sentence of the original version; this final sentence, which implied uncertainties in the fitted curves, is inappropriate in view of the corrected sampling rate.

The corrected Supplementary File 2 now includes the corrected calculations and presentations described above. The corrected Supplementary File 2 omits k_obs_ values for the lowest concentrations (0.245 μM) of iDianiSnFR and iCyt_F_SnFR; these traces were too noisy for systematic analysis.

We apologize to eLife readers, editors, and publication staff for these burdensome corrections. The corrections do not affect the paper’s major or minor conclusions. The corrections do not affect the detailed kinetic analysis of cytisine kinetics in Appendix 2.

